# Isolation and Quantification of Sphingosine and Sphinganine from Rat Serum Revealed Gender Differences

**DOI:** 10.3390/biom9090459

**Published:** 2019-09-07

**Authors:** Graham Brogden, Diab M. Husein, Pablo Steinberg, Hassan Y. Naim

**Affiliations:** 1 Department of Physiological Chemistry, University of Veterinary Medicine Hannover, Buenteweg 17, 30559 Hannover, Germany (D.M.H.) (H.Y.N.); 2 Max Rubner-Institute, Federal Research Institute of Nutrition and Food, Haid-und-Neu-Str. 9, 76131 Karlsruhe, Germany

**Keywords:** high performance liquid chromatography, sphingosine, sphinganine, serum

## Abstract

Sphingolipids are an important group of lipids that play crucial roles in living cells, facilitating cell recognition, signal transduction and endocytosis. The concentration of sphingosine and some of its derivatives like sphinganine may serve as a biomarker for the diagnosis of sphingolipidoses or be used for further research into similar diseases. In this study, a sphingolipid extraction and a high resolution detection method specific for sphingosine and sphinganine was adapted and tested. Lipids were extracted from rats’ serum, coupled to *o*-phthalaldehyde and detected with a fluorescence detector after running through a silica gel column in a high performance liquid chromatography system. With this method, we analysed 20 male and 20 female rat serum samples and compared the concentrations of sphingosine and sphinganine. The results showed a significant difference between the sphingosine concentrations in the male and female rats. The sphingosine concentration in female rats was 805 ng/mL (standard deviation, SD ± 549), while that in males was significantly lower at (75 ng/mL (SD ± 40)). Furthermore, the sphingosine:sphinganine ratio was almost 15-fold higher in the females’ samples. The method presented here facilitates the accurate quantification of sphingosine and sphinganine concentrations down to 2.6 ng and 3.0 ng, respectively, and their ratio in small amounts of rat serum samples to study the sphingolipid metabolism and its potential modulation due to gene mutations or the effect of prevalent toxins.

## 1. Introduction

**Figure 1:**
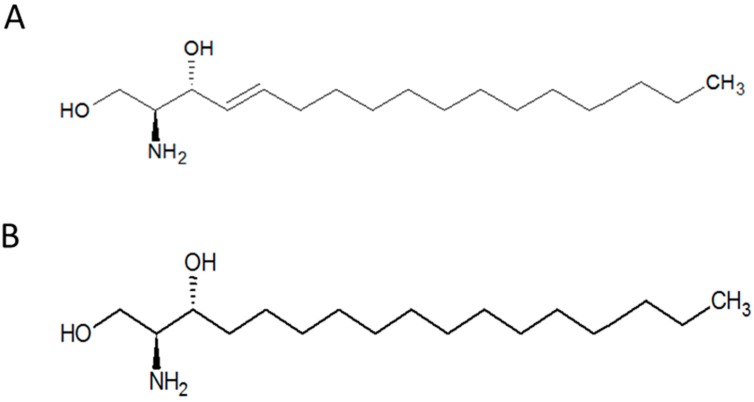
Structure of sphingosine (**A**) and sphinganine (**B**)**.**

Sphingosine (SPO) metabolites are highly bioactive compounds and are involved in diverse cellular processes, including cell–cell interaction, cell proliferation, differentiation and apoptosis. SPO Sphingosine is one of the primary components of sphingolipids, which are one of the most important amphiphilic lipid classes in the cell membrane [1]. This compound belongs to the class of organic compounds known as amino alcohols. SPO (Figure 1A) and its chemically modified forms, such as sphingosine-1-phosphate, serve as the backbone of some complex sphingolipids like ceramides and glycosphingolipids. Furthermore, phosphoethanolamine, a phospholipid precursor, can also be synthesised from sphingosine [2].

Sphingolipids play a critical role in neuronal tissues. Specific disorders in the metabolism of sphingolipids [3], such as lysosomal storage diseases including: Niemann–Pick disease, Fabry disease or Gaucher disease [4] and also compounds like fumonisin B1 [5] may disrupt the biosynthesis or metabolism of sphingolipids and cause the accumulation of their precursors, which may be detrimental for the cell.

Sphinganine (SPA, Figure 1B), a derivative of SPO, is an intermediate in the production of ceramide. This compound is biosynthesised in the Endoplasmic Reticulum, from palmitoyl-CoA and serine. SPA Sphinganine can be also modified, for instance, sphinganine kinase adds a phosphate group to C1 to produce sphinganine-1-P. This compound can be degraded so that some lipids, such as phosphoethanolamine, and selected fatty acids can be produced. Sphinganine and some of its synthetic derivatives like safingol, which is the L-threo-stereoisomer of endogenous sphinganine, play important roles in some cascade reactions and processes in living cells. Excess accumulation of SPO, for example, has been shown to inhibit the cholesterol transport in Niemann–Pick Type C (NPC) disease [6]. It can inhibit the esterification of cholesterol and causes unesterified cholesterol to accumulate in perinuclear vesicles, subsequently blocking post-lysosomal cholesterol transport [7].

In this study, a sensitive high-performance liquid chromatography (HPLC) fluorescence detection method for SPO and SPA is adapted to determine low concentrations of both lipids in rat serum. Furthermore, comparisons are made between the levels of SPA and SPO present in male and female rat serum.

## 2. Materials and Methods 

### 2.1. Animals

The animal study was approved by the Veterinary State Administration of the Slovak Republic and animal care was in compliance with the European Convention for the Protection of Vertebrate Animals used for Experimental and other Scientific Purposes. Moreover, the trial was performed in the experimental animal facility of the Department of Toxicology of the Slovak Medical University in Bratislava (Slovakia) in compliance with good laboratory practice (GLP) rules. Serum samples used in this study were taken as part of a previously published study [8]. Serum samples were taken from 20 male and 20 female 18-week-old Wistar Han RCC rats that were fasted for 16 h prior to blood sampling. Samples were subsequently stored at −20 °C. 

### 2.2. Sample Preparation

The lipid extraction protocol was based on Qui and Liu [9] and Hammad et al. [10], with minor alterations. One hundred microliters rat serum was pipetted into an 8 mL glass tube with a Teflon covered cap. We added 2 mL isopropanol:ethyl acetate (15:85 *v/v*) to the serum. The glass tube was vortexed for 30 s and centrifuged for 5 min at 3000× *g* (Heraeus, Hanau, Germany). The upper liquid phase was removed and transferred to a new glass tube. To the lower pellet phase, 100 µL formic acid (98%) and 2 mL isopropanol:ethyl acetate (15:85 *v/v*) were added. The tube was then vortexed for 30 s and centrifuged for 5 min at 2500× *g*. The upper liquid phase was removed and added to the initial extract. The extract was dried at 60 °C in a vacuum centrifuge. After drying, lipids were dissolved in 50 µL methanol. The lipids were derivatized using *o*-phthalaldehyde (OPA) and thereafter 50 µL OPA reagent (5 mg OPA dissolved in 95 µL ethanol (100%) and 5 µL 2-mercaptoethanol added to 9.9 mL (3%) boric acid in water adjusted with KOH to pH 10.5 were added, and the mixture was incubated for 5 min. Then, 900 µL methanol:5 mM potassium phosphate buffer (90:10 pH 7) was added to the sample, and the 1 mL volume was filtered into HPLC brown glass vials using a 1 mL syringe and a 0.25 µm sterile filter. 

### 2.3. Lipid Analysis

Sphingosine and Sphinganine were separated and quantified using a HPLC Chromaster system coupled to a fluorescence detector (Hitachi, Tokyo, Japan) with a 100–4.6 mm Chromolith HighResolution RP-18e column plus a RP-18 endcapped 5–4.6 mm guard cartridge (both Merck, Darmstadt, Germany). Methanol:5 mM potassium phosphate buffer pH = 7 (90:10 *v/v*) was used as a mobile phase at a flow rate of 1 mL/min. The samples were stimulated with an excitation light of 340 nm and the detection occurred at an emission wavelength of 455 nm using a Xenon lamp (Hitachi, Tokyo, Japan). Each run lasted 10 min and each sample was analysed in duplicate.

### 2.4. Statistical Analysis

Repeat measurements with a greater than 20% internal variance were excluded from the analysis. Statistical analysis was performed in Microsoft Excel 2010 and GraphPad Prism version 8. Outliers were removed using the ROUT test and the statistical analysis was performed using a one way t-test. The limit of quantification was calculated at three times the y-intercept for each lipid.

## 3. Results

**Figure 2:**
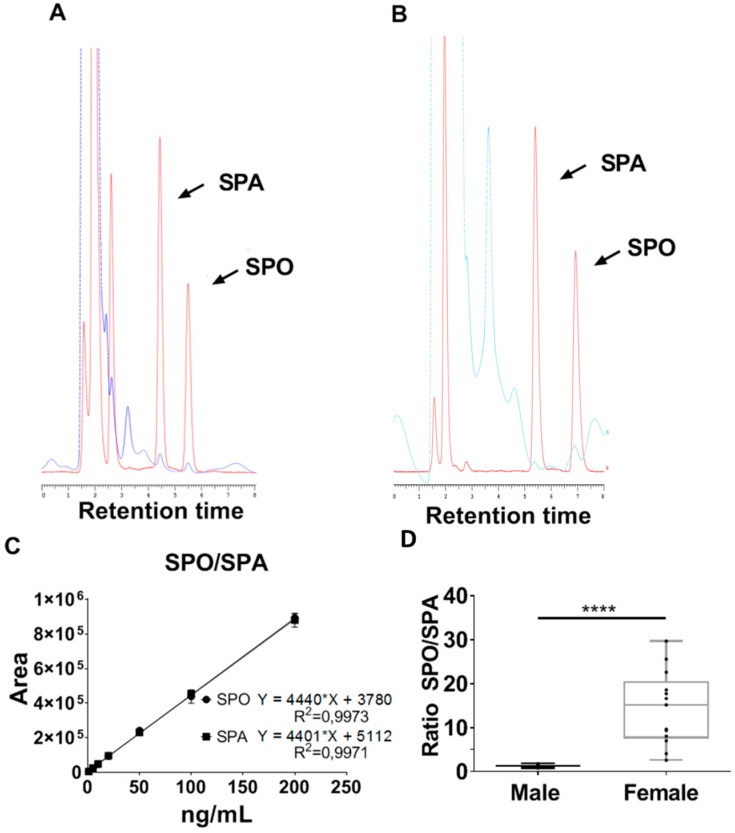
Chromatogram showing sphinganine and sphingosine with retention times of 4.6 min and 5.6 min in red, respectively, and a representative male serum sample in dark blue (**A**). A representative female serum sample shown in light blue, with sphinganine and sphingosine having a retention time of 5.5 min and 7.0 min, respectively. The standard is shown in red (**B**). Standard curves for sphinganine and sphingosine (**C**). The high-performance liquid chromatography (HPLC) analysis of sphingosine (SPO) and sphinganine (SPA) in serum samples from male and female rats. The results are presented as the ratio of sphingosine/sphinganine (**D**). Statistical analysis performed by a one-way t-test. **** *p* ≤ 0.0001, data shown are of at least 10 independent samples. Results are presented as standard error of the mean

Here we describe an improved method to separate and quantify SPO and SPA from rat serum samples using HPLC- coupled to a fluorescence detector. Negligible background levels were present and the signals emitted from OPA, SPO and SPA were separated and had retention times of 2.0, 4.5–5.5 and 5.5–7.0 min, respectively (Figure 2A–C). The identity of peaks was confirmed via the addition of either 25 ng SPO or SPA to samples prior to isolation and compared to untreated samples. An increase in the area under the peaks corresponded to the expected amounts detected when 25 ng of each lipid were analysed individually. Furthermore, no additional peaks were detected.

The detection limit of the method described here is approximately 1 ng, with the limit of quantification at 2.6 ng and 3.0 ng for sphingosine and sphinganine, respectively, and thus sufficient to quantify SPO and SPA in 100 µL serum.

Interestingly, the serum concentration ratio of SPO:SPA between male and female was significantly different, with male rats having a ratio of just over 1:1 and females 14:1 (Figure 2D). This difference is primarily due to the large increase in SPO serum levels detected in female rats. Analysis of male rat serum revealed 75 ng (SD ± 40) and 51 ng (SD ± 32) of sphingosine and sphinganine, respectively, whereas female serum contained 805 ng (SD ± 549) and 62 ng (SD ± 61) of sphingosine and sphinganine, respectively.

## 4. Discussion

The isolation protocol was based on a procedure previously described by Hammad et al. [10], which was designed for the analysis of sphingolipids in plasma. Ceramide and some of its derivatives were analysed, however, the products or precursors such as SPO and SPA were not. Using the method described here, we isolated and detected SPO and SPA and could reliably detect both lipids in low nanoscale concentrations, which is an improvement in comparison to the protocol of Hammad et al. [10]. Furthermore, the use of a fluorescence detector instead of mass spectrometry () reduces costs and the time taken to measure each sample. Methods using quadrupole-time-of-flight mass spectrometry are highly precise and sensitive, but they are also very expensive when compared to the method used in this study for the detection of sphingolipids, which can be sufficient for many studies [11].

Previous studies showed a detection limit at a picomolar scale for the SPO and SPA concentration in human urine [9]. In our study we analysed SPO and SPA in rats’ serum, which contains a higher concentration of lipids and proteins [12]. Although the method presented here is a low cost method, the sensitivity and reproducibility is comparable with other higher cost methods using HPLC-MS [13].

Gender associated differences between SPO, SPA or their ratio, although hypothesised, have not been described. A tendency for higher blood levels of sphingolipids has been previously observed in women [10] and female mice [14], the higher concentrations being attributed to the higher levels of oestrogen. Furthermore, 45-day-old male rats contained approximately 9 ng/mL sphingosine, with 3 ng sphinganine present, which is less than the values detected here in 18-week-old male rats [15]. This suggests sex- and potentially age-dependent differences in lipid metabolism and must be taken into account when using serum levels as a biomarker or when studying the roles of sphingolipids in diseases.

## 5. Conclusions

In summary, the method described here enables the separation and quantification of nanogram amounts of SPO and SPA in rat serum samples. Furthermore, significant differences in the concentration and metabolism of SPO and SPA were identified between male and female rats.
